# Effect of *Ascosphaera apis* Infestation on the Activities of Four Antioxidant Enzymes in Asian Honey Bee Larval Guts

**DOI:** 10.3390/antiox12010206

**Published:** 2023-01-16

**Authors:** Kaiyao Zhang, Zhongmin Fu, Xiaoxue Fan, Zixin Wang, Siyi Wang, Sijia Guo, Xuze Gao, Haodong Zhao, Xin Jing, Peiyuan Zou, Qiming Li, Mengjun Chen, Dafu Chen, Rui Guo

**Affiliations:** 1 College of Animal Sciences (College of Bee Science), Fujian Agriculture and Forestry University, Fuzhou 350002, China; 2 Apitherapy Research Institute, Fujian Agriculture and Forestry University, Fuzhou 350002, China

**Keywords:** *Ascosphaera apis*, chalkbrood, honey bee, *Apis cerana*, antioxidant enzyme, polyphenol oxidase, host response

## Abstract

*Ascosphaera apis* infects exclusively bee larvae and causes chalkbrood, a lethal fungal disease that results in a sharp reduction in adult bees and colony productivity. However, little is known about the effect of *A. apis* infestation on the activities of antioxidant enzymes in bee larvae. Here, *A. apis* spores were purified and used to inoculate Asian honey bee (*Apis cerana*) larvae, followed by the detection of the host survival rate and an evaluation of the activities of four major antioxidant enzymes. At 6 days after inoculation (dpi) with *A. apis* spores, obvious symptoms of chalkbrood disease similar to what occurs in *Apis mellifera* larvae were observed. PCR identification verified the *A. apis* infection of *A. cerana* larvae. Additionally, the survival rate of larvae inoculated with *A. apis* was high at 1–2 dpi, which sharply decreased to 4.16% at 4 dpi and which reached 0% at 5 dpi, whereas that of uninoculated larvae was always high at 1~8 dpi, with an average survival rate of 95.37%, indicating the negative impact of *A. apis* infection on larval survival. As compared with those in the corresponding uninoculated groups, the superoxide dismutase (SOD) and catalase (CAT) activities in the 5- and 6-day-old larval guts in the *A. apis*–inoculated groups were significantly decreased (*p* < 0.05) and the glutathione S-transferase (GST) activity in the 4- and 5-day-old larval guts was significantly increased (*p* < 0.05), which suggests that the inhibition of SOD and CAT activities and the activation of GST activity in the larval guts was caused by *A. apis* infestation. In comparison with that in the corresponding uninoculated groups, the polyphenol oxidase (PPO) activity was significantly increased (*p* < 0.05) in the 5-day-old larval gut but significantly reduced (*p* < 0.01) in the 6-day-old larval gut, indicating that the PPO activity in the larval guts was first enhanced and then suppressed. Our findings not only unravel the response of *A. cerana* larvae to *A. apis* infestation from a biochemical perspective but also offer a valuable insight into the interaction between Asian honey bee larvae and *A. apis*.

## 1. Introduction

Honey bees are of great importance thanks to their pollination of numerous wildflowers and agricultural crops; their production of api-products, such as honey, royal jelly, propolis, beeswax, and bee pollen; and their scientific applications as research models [1]. However, as a kind of representative eusocial insect, honey bees are prone to infections by various pathogens and parasites, such as bacteria, fungi, viruses, and Varroa mite [2,3]. Among these, *Ascosphaera apis*, an obligate fungal pathogen of honey bee broods, causes chalkbrood disease and results in a dramatic decline in colony strength and productivity alone or in combination with other biotic or abiotic factors, which has given rise to severe losses for the apicultural industry [4]. For insects, including honey bees, the midgut is not only a pivotal tissue responsible for food digestion, enzyme secretion, and nutrient absorption but also a primary site for interactions between bee hosts and pathogenic microorganisms [5].

The fungal pathogen invades the body wall and then multiplies in the blood cavity, leading to the death of the host. After long-term interaction and coevolution, insects have evolved various mechanisms to resist pathogenic infestation, including physical and chemical barriers and an innate immune system [6]. Insects such as honey bees can produce reactive oxygen species (ROS), a byproduct of their aerobic metabolism, to defend themselves against xenobiotics or pathogens, thus maintaining the stability of their internal environment [6]. All aerobic organisms, including insects, have specific antioxidant systems to scavenge excess oxygen radicals in the body to avoid oxidative damage [7]. Studies have shown that organisms protect themselves from oxidative damage by regulating intracellular ROS production through a system of antioxidant enzymes, including superoxide dismutase (SOD), catalase (CAT), and glutathione S-transferase (GST) [8,9,10,11,12,13]. Accumulating evidence has suggested that antioxidant enzymes are employed by insects as weapons to combat pathogen invasion. For instance, Karthi et al. [14] reported that *Aspergillus flavus* was capable of infecting *Spodoptera litura* by directly acting on the host’s immune system, giving rise to a significant increase in SOD activity and a significant reduction in PO activity in *S. litura* larvae at higher conidial concentrations following exposure to *A. flavus*. Li et al. [15] observed that the *Heterorhabditis beicherriana* infection induced oxidative stress in *Tenebrio molitor* larvae, which resulted in larval antioxidative responses; the SOD activity in larvae treated with higher doses significantly increased at 24 h after infection, whereas the activities of POD and CAT significantly decreased at 32 h after infection. Shi et al. [16] detected that the ROS content and antioxidant enzyme activity significantly increased in *Bombyx mori* at 72 or 96 h after BmNPV infection, indicating that the antioxidant enzyme activity was activated at the later stage of viral infection. Recently, Li et al. [10] reported that *A. apis* infestation induced oxidative stress in western honey bee (*Apis mellifera*) worker larvae, resulting in a significant decrease in the enzymatic activities of SOD, CAT, and GST. However, owing to a lack of related studies, whether *A. apis* infestation affects the activities of major antioxidant enzymes in Asian honey bees (*Apis cerana*), a sister species of *A. mellifera*, has been largely unknown until now.

*A. cerana*, a bee species distributed throughout various climatic zones of Asia, is predominant and widely used in the Asian beekeeping industry, and it plays a critical role in local pollination and ecological maintenance [17,18]. In the beekeeping practice, *A. mellifera* colonies are commonly liable to *A. apis* infection, and *A. mellifera* larvae often die from chalkbrood disease. Previous studies have focused mainly on interactions between western honey bee (*Apis mellifera*) larvae and *A. apis* [4,19]. The preliminary results of Tejerina et al. [20] demonstrated that *Lactobacillus* spp. prevent *A. mellifera* larvae from mummifying or forming sporocysts on the surface when used against some Spanish and Argentine *A. apis* strains. Ye et al. [21] showed that DElncRNAs potentially participated in the *Apis mellifera ligustica* larval immune response to *A. apis* invasion by regulating the expression of neighboring genes or interacting with DEmiRNAs.

Over the past few years, our group has conducted an array of molecular and omics studies on interactions between *A. cerana* larvae and *A. apis*. First, Chen et al. [22] isolated *A. apis* from chalkbrood mummies of *A. cerana* drone larvae and offered the first morphological and molecular evidence of this, followed by the verification of the *A. apis* infection of the *A. cerana* worker larvae by inoculation with *A. apis* spores under lab conditions. Second, Guo et al. [23] deciphered the cellular and humoral immune responses of *A. cerana* larvae to *A. apis* invasion on the basis of next-generation sequencing and transcriptome investigation; we analyzed the *A. cerana* 6-day-old larval response to *A. apis* infestation mediated by noncoding RNAs, including miRNAs, lncRNAs, and circRNAs [24,25,26]. Third, Guo et al. [27] investigated the transcriptomic dynamics of *A. apis* infecting *A. cerana* larvae and the potential mechanism underlying fungal infection. Fourth, Xiong et al. [24] analyzed the expression profile and regulatory network of miRNAs in *A. apis* invading *A. cerana* 6-day-old larva and further elucidated the miRNA-regulated mechanism of *A. apis* invasion. However, biochemical research regarding *A. cerana*-*A. apis* interaction is very limited at present, hindering a deeper understanding of the mechanism underlying responses of *A. cerana* to *A. apis* infestation.

In the current work, *A. apis* spores were purified and used to inoculate the *A. cerana* 3-day-old larvae, followed by a calculation of the host survival rate and an evaluation of the activities of four antioxidant enzymes, specifically SOD, CAT, GST, and polyphenol oxidase (PPO), in the guts of both *A. apis*–inoculated and uninoculated larvae. The findings from the present study could not only clarify the effect of *A. apis* invasion on the host survival rate and the activities of the aforementioned four antioxidant enzymes of great importance in larval guts but also offer a valuable understanding of *A. cerana* larval responses to *A. apis* and host–pathogen interactions.

## 2. Materials and Methods

### 2.1. Fungi and Bee Larvae

*A. apis* was previously isolated from chalkbrood mummies and conserved at the Honey Bee Protection Laboratory of the College of Animal Sciences (College of Bee Science), Fujian Agriculture and Forestry University, Fuzhou, China [2,28,29]. *A. cerana* worker larvae were derived from 3 strong colonies reared in the apiary of the College of Animal Sciences (College of Bee Science), Fujian Agriculture and Forestry University, Fuzhou, China.

### 2.2. Purification of A. apis Spores

*A. apis* stored at 4 °C was transferred to PDA medium and cultured at 33 ± 0.5 °C in a constant temperature and humidity chamber (Jingke, Shanghai, China). After 10 days of culturing, white mycelia were removed, and black fruiting bodies were harvested and transferred to an RNAase-free EP tube, following our previously established protocol [30,31]. Next, 1 mL of sterile water was added to the EP tube, followed by complete grinding. Then, the grinding fluid was centrifuged at 25 °C, 7000× *g* for 3 min. The supernatant was removed, and 1 mL of sterile water was added, followed by centrifugation at 25 °C, 7000× *g* for 3 min. The centrifugation was repeated twice to clean the spores, which were then stored at 4 °C until use.

### 2.3. Experimental Inoculation and Survival Rate Calculation

Honey bee larvae were reared and inoculated with *A. apis* spores by following our previously described method [23]. In brief, (1) the larvae diet was prepared following Feng et al. [32], preheated to 35 °C, and added to 6-well culture plates. (2) PCR amplification was performed to detect the honey bee colonies reared in the apiary, and three colonies with negative results were selected as experimental colonies. The 2-day-old larvae were carefully transferred to 6-well culture plates using a Chinese graft, and after 24 h, the larvae were transferred to 48-well culture plates (1 larva/well) and placed in an incubator (35 °C, 95% RH). (3) The purified *A. apis* spores were subjected to gradient dilution and mixed with the diet, with a final concentration of 1 × 10^7^ spore/mL. The 3-day-old treatment group larvae were fed the diet containing spores (0 days after inoculation, 0 dpi), while 3-day-old larvae in the control groups were fed the diet without spores; the diet was changed daily. There were 3 biological replicas of this experiment. The dead larvae in both treatment and control groups were recorded and removed every 24 h until 8 dpi, followed by survival rate calculations.

The *A. apis*–inoculated 4-, 5-, and 6-day-old larvae guts (AcT1, AcT2, and AcT3 groups, respectively) as well as the uninoculated 4-, 5- and 6-day-old larval guts (AcCK1, AcCK2, and AcCK3 groups, respectively) were respectively dissected following our previously established method [23], frozen in liquid nitrogen, and kept at −80 °C until use.

### 2.4. Evaluation of the SOD Activity

Following the method described by Li et al. [10], the larval gut SOD activity was evaluated using the Insect SOD ELISA Kit (MLBIO, Shanghai, China). Briefly, the gut samples were fully ground with a high-throughput tissue grinder (MEIBI, Hangzhou, China); the grinding fluid was transferred to a sterile EP tube; 750 mL of 1 × PBS solution was added, followed by centrifugation at 1000× *g* for 10 min; the supernatant was incubated on ice; 50 mL of the standard sample was added to the standard well, while 40 mL of sample diluent and 10 mL of grinding fluid were added into the sample well, followed by gentle shaking. Meanwhile, a blank well was set and sealed with a film, and the microtiter plate was incubated at 37 °C for 30 min. The reaction solution was then discarded, and washing buffer was added to the wells. The solution was removed after standing for 30 s, and the operation was repeated 5 times. Then 50 mL of enzyme standard reagent was added to each standard well and sample well, and 50 mL each of chromogenic A and B were added. After gentle shaking, the solution was placed at 37 °C for 10 min; 50 mL of termination solution was added into each well to terminate the reaction; finally, the OD value at 450 nm from each well was measured by a Thermo Scientific Varioskan LUX (ThermoFisher, Waltham, MA, USA). This experiment included three biological replicas.

The specific antioxidant enzyme activity was expressed as units of enzyme activity per milligram of protein. Data were analyzed and plotted by GraphPad Prism 8 software (GraphPad Software, San Diego, CA, USA). Experimental data were presented as mean ± SD and subjected to Student’s *t*-tests.

### 2.5. Examination of CAT and GST Activities

According to the method described by Li et al. [10], the larval gut CAT activity was examined with an Insect CAT ELISA Kit (MLBIO, Shanghai, China). GST activity was checked using an Insect GST ELISA Kit (MLBIO, Shanghai, China) by following the described method by Li et al. [10]. The operation and calculation methods were the same as in Section 2.4.

### 2.6. Detection of PPO Activity

On basis of the method described by Li et al. [33], the larval gut PPO activity was evaluated using the Polyphenol Oxidase (PPO) Activity Assay Kit (Solarbio, Beijing, China). The gut samples (each 0.1 g) in the six groups mentioned above were transferred to sterile EP tubes, and 1 mL of extraction solution (0.05 M sodium phosphate (pH 7.0), 4% (*w/v*) insoluble PVP, and 0.5% (*w/v*) Triton X-100) was added. Next, the gut tissues were thoroughly ground using a high-throughput tissue grinder, followed by centrifugation at 8000× *g* for 10 min. The supernatant was transferred to a new EP tube and placed on ice for measurement. The assay tube and control tube reaction systems were prepared, placed in a 25 °C water bath for 10 min, and then quickly transferred to a 100 °C metal bath for 10 min. The reaction system was mixed thoroughly and centrifuged at 5000× *g* for 10 min, and the supernatant was transferred to a new EP tube and placed on ice. The assay and control OD values were detected at 410 nm and respectively named A assay and A control, and the difference between them was named ΔA. PPO activity was calculated as follows: PPO (U/g) = 120 × ΔA ÷ W (ΔA = A assay − A control), where W is the sample mass in grams.

## 3. Results

### 3.1. Verification of A. cerana Larvae Infection by Inoculation with A. apis Spores

**Figure 1:**
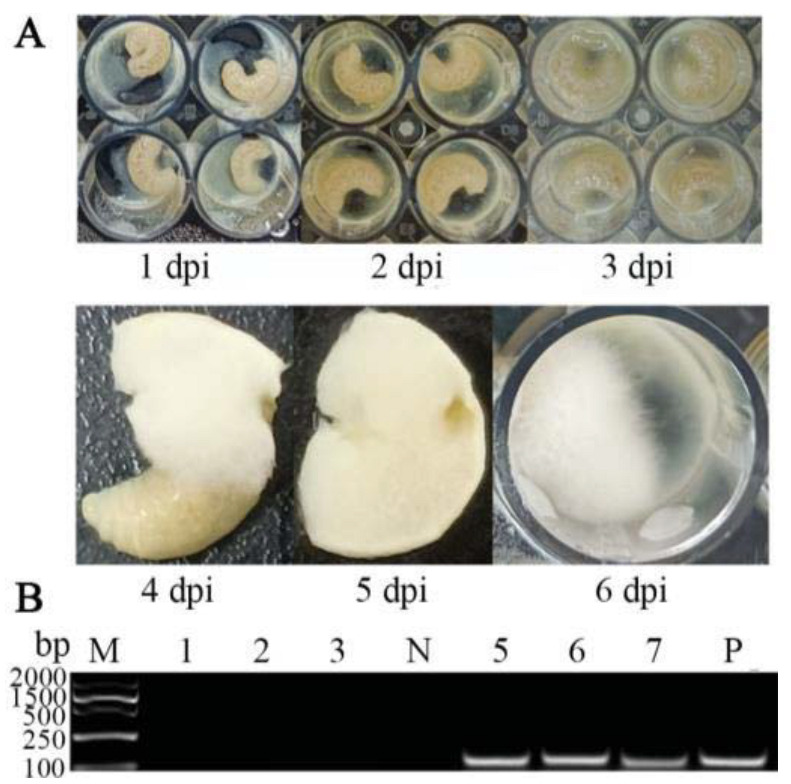
Observation and verification of *A. apis* infection of *A. cerana* larvae. (**A**) Observation of larval chalkbrood symptom after *A. apis* spore inoculation; (**B**) PCR validation of larval guts inoculated with *A. apis* spores. Lane M: DNA marker; Lane 1–3: uninoculated 6-, 5-, and 4-day-old larval guts; Lane N: sterile water (negative control); Lane 5–7: *A. apis*–inoculated 6-, 5-, and 4-day-old larval guts; Lane P: purified spores of *A. apis* (positive control).

In the present study, a prominent symptom of chalkbrood disease was observed in larvae inoculated with *A. apis* spores—white mycelia first penetrated from the posterior end of the larva at 4 dpi, extended to the anterior end, and eventually covered the entire larval body surface (Figure 1A). In addition, as shown in Figure 1B, agarose gel electrophoresis indicated that fragments with the expected sizes (about 217 bp) could be amplified from the *A. apis*–inoculated larval guts and *A. apis* spores but could not be amplified from the uninoculated larval guts and sterile water. Collectively, these results confirmed the infection of *A. cerana* larvae by inoculation with *A. apis* spores.

### 3.2. Survival Rate of A. cerana Larvae after A. apis Spores Infection

**Figure 2:**
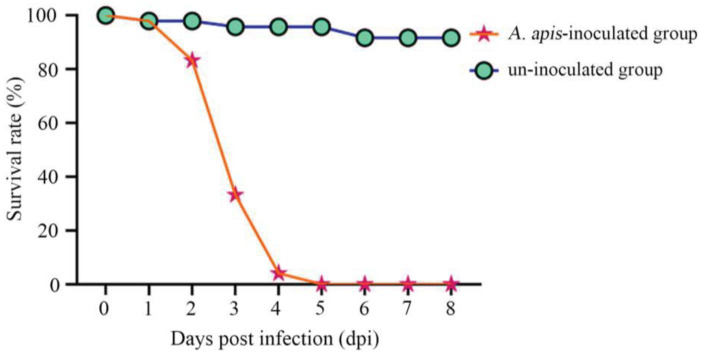
Survival rates of *A. apis*–infected and uninfected *A. cerana* larvae.

It was detected that the survival rate of *A. cerana* larvae in the *A. apis*–inoculated group was 97.92%, 83.33%, and 33.33% at 1 dpi–3 dpi, respectively; additionally, the larval survival rate sharply decreased to 4.16% at 4 dpi and to 0 at 5 dpi (shown in Figure 2). Comparatively, the survival rate of larvae in the uninoculated group was always high at 1 dpi–8 dpi, with an average survival rate of 95.37%, as shown in Figure 2.

### 3.3. Effect of A. apis Infection on SOD Activity in A. cerana Larval Guts

**Figure 3:**
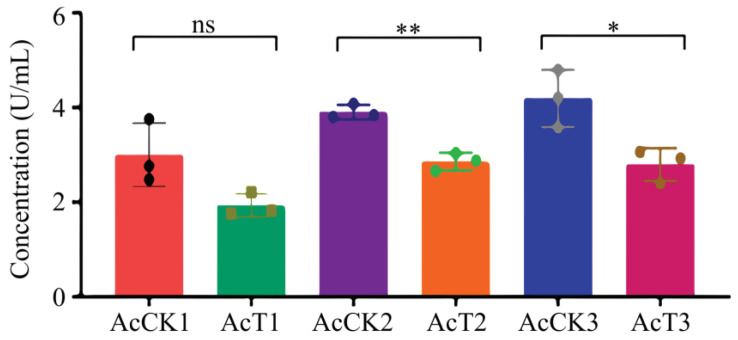
SOD activity in *A. cerana* 4-, 5-, and 6-day-old larval guts infected by *A. apis*. The experimental data are shown as mean ± SD and were subjected to Student’s *t*-tests, ns: *p* > 0.05, *: *p* < 0.05, **: *p* < 0.01. AcCK1, AcCK2, and AcCK3 respectively represent the uninfected 4-, 5-, and 6-day-old larval guts, whereas AcT1, AcT2, and AcT3 respectively represent the *A. apis*–infected 4-, 5-, and 6-day-old larval guts.

It was found that as compared with that in the corresponding uninfected groups, the SOD activity was reduced (*p* > 0.05) in the 4-day-old (1.93 ± 0.54 U/mL) larval gut, whereas significantly decreased (*p* < 0.05) in the 5- (2.86 ± 0.15 U/mL) and 6-day-old (2.79 ± 0.28 U/mL) larval guts in the *A. apis*–infected groups (Figure 3).

### 3.4. Effect of A. apis Infection on the CAT Activity in the A. cerana Larval Guts

**Figure 4:**
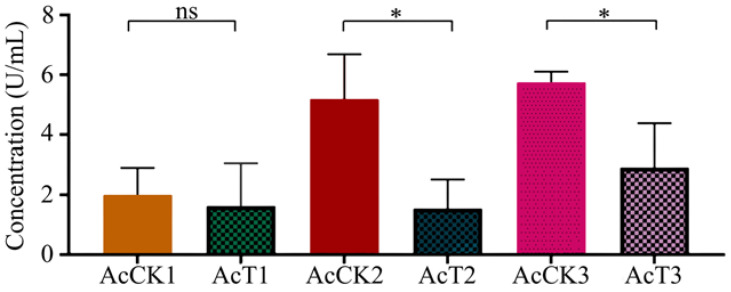
CAT activity in *A. cerana* 4-, 5-, and 6-day-old larval guts infected by *A. apis*. The experimental data are shown as mean ± SD and were subjected to Student’s *t*-tests, ns: *p* > 0.05, *: *p* < 0.05. AcCK1, AcCK2, and AcCK3 respectively represent the uninfected 4-, 5-, and 6-day-old larval guts, whereas AcT1, AcT2, and AcT3 respectively represent the *A. apis*–infected 4-, 5-, and 6-day-old larval guts.

Here, in comparison with that in the corresponding uninfected groups, the CAT activity was decreased (*p* > 0.05) in the 4-day-old (1.63 ± 1.17 U/mL) larval gut, whereas significantly reduced (*p* < 0.05) in the 5- (1.54 ± 0.79 U/mL) and 6-day-old (2.91 ± 1.20 U/mL) larval guts in the *A. apis*–infected groups (Figure 4).

### 3.5. Effect of A. apis Infection on GST Activity in A. cerana Larval Guts

**Figure 5:**
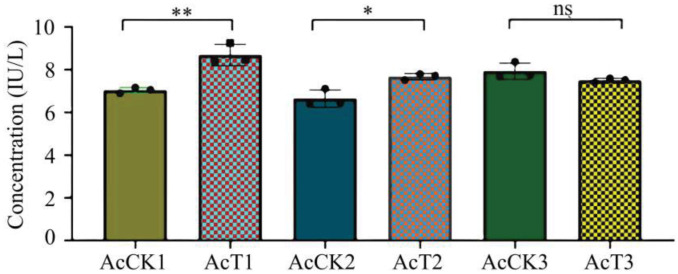
GST activity in *A. cerana* 4-, 5-, and 6-day-old larval guts infected by *A. apis*. The experimental data are shown as mean ± SD and were subjected to Student’s *t*-tests, ns: *p* > 0.05, *: *p* < 0.05, **: *p* < 0.01. AcCK1, AcCK2, and AcCK3 respectively represent the uninfected 4-, 5-, and 6-day-old larval guts, whereas AcT1, AcT2, and AcT3 respectively represent the *A. apis*–infected 4-, 5-, and 6-day-old larval guts.

In this current work, as compared with that in the corresponding control groups, the GST activity was significantly increased (*p* < 0.05) in the 4-day-old (8.69 ± 1.40 IU/L) and 5-day-old (7.67 ± 0.12 IU/L) larval guts, whereas reduced (*p* > 0.05) in the 6-day-old (7.50 ± 0.08 IU/L) larval gut (Figure 5).

### 3.6. Effect of A. apis Infection on PPO Activity in A. cerana Larval Guts

**Figure 6:**
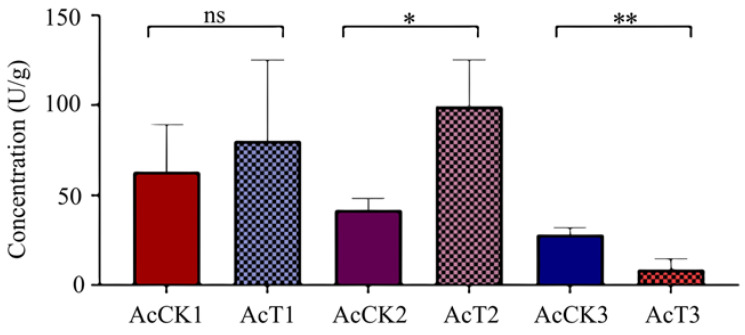
PPO activity in *A. cerana* 4-, 5-, and 6-day-old larval guts infected by *A. apis*. The experimental data are shown as mean ± SD and were subjected to Student’s *t*-tests, ns: *p* > 0.05, *: *p* < 0.05, **: *p* < 0.01. AcCK1, AcCK2, and AcCK3 respectively represent the uninfected 4-, 5-, and 6-day-old larval guts, whereas AcT1, AcT2, and AcT3 respectively represent the *A. apis*–infected 4-, 5-, and 6-day-old larval guts.

It was observed that in comparison with that in the corresponding control groups, the PPO activity was increased (*p* > 0.05) in the 4-day-old (80.08 ± 36.89 U/g) larval gut, significantly increased (*p* < 0.05) in the 5-day-old (99.27 ± 21.24 U/g) larval gut, and significantly decreased (*p* < 0.01) in the 6-day-old (8.74 ± 4.85 U/g) larval gut, as shown in Figure 6.

## 4. Discussion

Previously, there was no documentation of the course of chalkbrood disease that occurred in *A. cerana* larvae. It was reported that *A. apis* spores were consumed by *A. mellifera* larvae via food sharing and germinated in the midgut lumen, with the disappearance of the diaphragm between the midgut and hindgut at the prepupal stage [34]; the fungal spores and food debris then swarmed into the hindgut lumen, where the mycelia rapidly grew in contact with O_2_, followed by the penetration of the peritrophic membrane, gut wall, and body wall, eventually covering the entire larva, with a thick layer of white mycelia [4,24]. In the current work, the *A. apis* spores were purified and mixed with the diet to feed 3-day-old larvae of *A. cerana*, and no apparent symptoms of chalkbrood disease were detected at 1–3 dpi; however, mycelia penetrated from the posterior end of the larva at 4 dpi and then extended to the anterior end, finally covering the entire larval body surface (Figure 1A). This course of chalkbrood disease was similar to that of *A. mellifera* larvae infected by *A. apis* [35], which was in line with the fact that *A. apis* was an exclusive fungal pathogen of bee larvae. In addition, agarose gel electrophoresis showed that fragments of expected sizes (about 217 bp) could be amplified from *A. apis*–inoculated larval guts and *A. apis* spores, but not from the uninoculated larval guts and sterile water (Figure 1B). In summary, these results together confirmed the *A. apis* infection of *A. cerana* larvae after spore inoculation under lab conditions, which gave rise to chalkbrood disease. This offered solid experimental evidence for further study on interactions between *A. cerana* larvae and *A. apis*.

In the present study, we observed that the survival rate of *A. cerana* larvae after *A. apis* inoculation was 97.92%, 83.33%, 33.33% at 1~3 dpi, which sharply decreased to 4.16% at 4 dpi and to 0 at 5 dpi, whereas that of the uninoculated larvae was always high at 1~8 dpi (95.37% on average) (Figure 2). This indicated that the increased infection time of *A. apis* negatively influenced larval survival, following the pathogenesis mentioned above. To the best of our knowledge, this is the first experimental evidence of the survival rate of *A. cerana* larvae infected by *A. apis*.

In insects, protective and detoxifying enzymes are critical in maintaining normal physiological functions and biochemical metabolisms [8,10,36]. Among these, SOD and CAT can remove oxidative and toxic molecules such as O^2-^ and hydroxyl radical OH^-^ produced by exogenous compounds [11]. Li et al. [10] reported that both SOD and CAT activities were significantly reduced in the *A. m. ligustica* 3-day-old worker larvae at 96 h after inoculation with *A. apis* spores. In this current work, we found that as compared with those in the corresponding uninfected groups, the SOD and CAT activities in the 5- and 6-day-old *A. apis*–infected larval guts were significantly decreased, as shown in Figure 3 and Figure 4, similar to the finding in the *A. m. ligustica* larvae infected by *A. apis* [10]. However, the SOD and CAT activities in the 4-day-old larval guts were reduced, but there was no significant difference between the *A. apis*–infected and uninfected groups (Figure 3 and Figure 4), showing little influence from the two aforementioned antioxidant enzymes given that there was only low-level spore germination and mycelial growth in the early stage of *A. apis* infection. Collectively, these results demonstrated that *A. apis* infestation could negatively influence the SOD and CAT activities of honey bee larvae, which may be a strategy adopted by *A. apis* during the long-term coevolution and interactions with *A. cerana* larvae. After feeding the fourth- and fifth-instar larvae of *Hyphantria cunea* with the leaves of *Bacillus thuringiensis* transgenic poplar, Ding et al. [37] detected that the activities of both SOD and CAT displayed an increase–decrease trend. After inoculating *Nilaparvata lugens Stål* with *Metarhizium flavoviride* spores, Zhang et al. [36] found that the SOD and CAT activities continuously increased as infection time prolonged. Taken together, these results indicated that the SOD and CAT activities exhibited different trends in various insects responding to pathogen infections.

As a key component of the antioxidant enzyme system, GST is involved in the detoxification process of exogenous compounds in honey bees [12,13]. Yan et al. [38] discovered that infection by highly pathogenic strains of entomo pathogenic nematode significantly altered the GST activity in the *Anoplophora glabripennis* larvae, presenting an overall increase–decrease–increase trend. Huang et al. [39] observed that the GST activity was significantly higher in *Plutella xylostella* L. parasitized by *Diadegma semiclausum* than that in the control group. In the present study, we found that the GST activity in 4- and 5-day-old larval guts after *A. apis* infestation was significantly increased, as shown in Figure 5. Thus, *A. cerana* larvae were likely to enhance GST activity in response to the oxidative stress caused by *A. apis* infestation. However, it was detected that the GST activity in 6-day-old larval guts after *A. apis* infestation was reduced, without a significant difference between the *A. apis*–infected and uninfected groups (Figure 5), to a certain extent reflecting complex host–pathogen interaction and *A. apis*-caused attenuation of the GST activity.

In insects, PPO is engaged in melanin formation, keratinization, and wound healing and also exerts a pivotal function in host immune defense [40,41]. The activity and the content of PPO are often used as indicators for evaluating insect immunity [36,41]. Li et al. [42] documented that the PPO activity in infected *Apriona germari* larvae hemolymph increased to a maximum at 2.5 days after *Beauveria bassiana* infection and then decreased at 3 days after infection. Wertheim et al. [43] reported that the expression level of the *PPO3* gene in *Drosophila* was significantly upregulated at 48–72 h after the parasitic wasp challenge. In *Nilaparvata lugens Stål*, there was a significant elevation of PO content at 72 h after infection with *Metarhizium flavoviride* [36]. In this work, we observed increased PPO activity in the 4-day-old larval gut, which was significantly elevated in the 5-day-old larval gut after *A. apis* infestation. The results showed that with the accumulation of spores and mycelia in the larval gut, the host reinforced the PPO activity to combat the *A. apis* infestation. Intriguingly, the PPO activity was significantly decreased in the gut tissue of 6-day-old larvae infected by *A. apis* (Figure 6). This indicated that at the late stage of infection, the PPO activity in the larval gut was suppressed because of the growing fungal stress. Taken together, these findings were suggestive of complex interactions between *A. cerana* larvae and *A. apis*.

## 5. Conclusions

**Figure 7:**
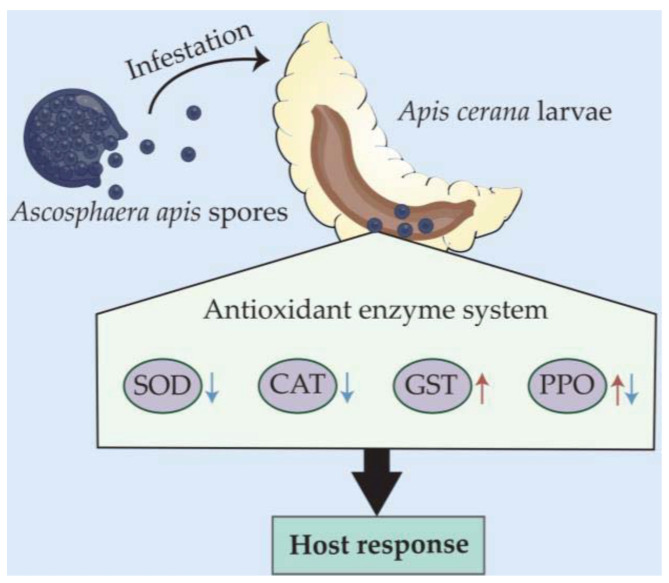
A hypothetical schematic diagram of the effect of *A. apis* infestation on the activities of four antioxidant enzymes in *A. cerana* larval guts.

In a nutshell, the *A. apis* spore inoculation of *A. cerana* larvae gave rise to chalkbrood disease, which decreased the host survival rate. The *A. apis* infestation affected the activities of SOD, CAT, GST, and PPO (Figure 7), indicative of a host-adopted defense strategy mediated by antioxidant enzymes and complex host–pathogen interactions.

## Data Availability

All the data is contained within the article.
